# Mining genome traits that determine the different gut colonization potential of *Lactobacillus* and *Bifidobacterium* species

**DOI:** 10.1099/mgen.0.000581

**Published:** 2021-06-08

**Authors:** Yue Xiao, Jianxin Zhao, Hao Zhang, Qixiao Zhai, Wei Chen

**Affiliations:** ^1^​ State Key Laboratory of Food Science and Technology, Jiangnan University, Wuxi, Jiangsu 214122, PR China; ^2^​ School of Food Science and Technology, Jiangnan University, Wuxi, Jiangsu 214122, PR China; ^3^​ National Engineering Research Center for Functional Food, Jiangnan University, Wuxi, Jiangsu 214122, PR China; ^4^​ Yangzhou Institute of Food Biotechnology, Jiangnan University, Yangzhou, Jiangsu 225004, PR China; ^5^​ Wuxi Translational Medicine Research Center and Jiangsu Translational Medicine Research Institute Wuxi Branch, Wuxi 214122, PR China; ^6^​ International Joint Research Laboratory for Probiotics at Jiangnan University, Wuxi, Jiangsu 214122, PR China

**Keywords:** comparative genomics, gut colonization, *Lactobacillus*, Bifidobacterium, engraftment prediction, lifestyle

## Abstract

Although the beneficial effects of probiotics are likely to be associated with their ability to colonize the gut, little is known about the characteristics of good colonizers. In a systematic analysis of the comparative genomics, we tried to elucidate the genomic contents that account for the distinct host adaptability patterns of *Lactobacillus* and *Bifidobacterium* species. The *Bifidobacterium* species, with species-level phylogenetic structures affected by recombination among strains, broad mucin-foraging activity, and dietary-fibre-degrading ability, represented niche conservatism and tended to be host-adapted. The *Lactobacillus* species stretched across three lifestyles, namely free-living, nomadic and host-adapted, as characterized by the variations of bacterial occurrence time, guanine–cytosine (GC) content and genome size, evolution event frequency, and the presence of human-adapted bacterial genes. The numbers and activity of host-adapted factors, such as bile salt hydrolase and intestinal tissue-anchored elements, were distinctly distributed among the three lifestyles. The strains of the three lifestyles could be separated with such a collection of colonization-related genomic content (genes, genome size and GC content). Thus, our work provided valuable information for rational selection and gut engraftment prediction of probiotics. Here, we have found many interesting predictive results for bacterial gut fitness, which will be validated *in vitro* and *in vivo*.

## Data Summary

All of the genome sequences used in this study are publicly available in the National Center for Biotechnology Information (NCBI) database; please see Table S1 (available with the online version of this article) for detailed information.

**Impact Statement**

Because little is known about the characteristics of good colonists, in this study we present a comprehensive mining effort to decode the hidden traits, combining both targeted and untargeted population genomic analysis, to guide the rational selection and gut engraftment prediction of probiotics. We focus on the whole picture of lifestyle separation of *Lactobacillus* and *Bifidobacterium* species, their phylogenetic features, characterization of carbohydrate profiles, distribution of intestinal tissue-anchored structures, phylotypes, activity and copy numbers of bile salt hydrolases (BSHs), and presence and absence of human-specific bacterial genes. We demonstrate that the strains of the three lifestyles can be separated with a collection of colonization-related genomic contents, and by detecting the presence or absence of specific genes, the natural niche of bacteria can be proposed. Given the limitations of the current gut colonization evaluation approach and the ineffectiveness of the current method for the determination of the natural niche of bacteria, the genome contents mined in this work might provide an alternative and/or supplementary means of predicting bacterial engraftment or their niche in nature.

## Introduction

*Lactobacilli* and *Bifidobacteria* are Gram-positive bacteria that are found in nutrient-rich habitats, including traditional fermented food, plants, silage, insects, mammals and humans. An increasing body of evidence shows that some members of these species, known as probiotics, can exhibit beneficial effects by modulating the indigenous microbiota [1] and immune system [2, 3], regulating crucial pathways in epithelial cells [4–6] and protecting gut barrier function [7, 8]. The question of whether distinct *Lactobacillus* and *Bifidobacterium* strains show different gut fitness in both a species-specific and a strain-specific manner has been widely investigated [9–14]. There is some evidence that *Bifidobacterium longum* AH1206, which was able to initialize and persist stably in the gut for more than 6 months after 2 weeks of oral administration, can interact more closely with the host microbiome and thus exert health-promoting effects [15]. In a recent review trying to summarize the relationship between gut colonization ability of probiotic strains and their health-promoting functions, Duar *et al*. proposed that where the long-term effects and metabolic activity of these strains are essential or important, gut colonization ability should be emphasized (i.e. strains can show lasting functionality and modulate microbial ecology in the gut after a single dose); otherwise, when immunoregulatory impacts are needed, strains that cannot initiate establishment in the gut might provoke stronger stimulation to the host immune system (strains with strong immunogenicity can be eradicated by the host immune defence) [16]. Although studies have identified some bacterial genes, such as pili, luxS and bile salt hydrolase (BSH), as being important in host–microbe interactions [17–20], reliable parameters that predict the potential for gut colonization by bacterial strains have not yet been defined. Such knowledge can improve the informed use of probiotics in resolving many health issues.

A common approach to evaluate gut adaptability involves the detection of strain shedding in the faeces to understand the establishment and persistence of ingested *Lactobacillus* and *Bifidobacterium* strains in the gut [12, 15]. Whereas this is, of course, a useful strategy, obtaining the colonization data for all available *Lactobacillus* or *Bifidobacterium* strains is impractical. In addition, the experimental settings of these previous studies are abstracted from any natural history of the strains. Indeed, the bacterial species are generally allochthonous to the studied hosts and cannot engraft in the gut niche [21–23], misleading us to draw a paradoxical conclusion that most of available probiotic strains are ‘passers-by’ and can only colonize in the gut transiently [22]. Here, based on ecological and evolutionary perspectives, we decided to adopt a different approach. Although the horizontal transmission of strains across geographical locations and hosts is generally accepted [24, 25], we postulated that the statistical data on frequently isolated origins of *Lactobacillus* and *Bifidobacterium* species are still representative of their niches and their colonization potential in the corresponding environments.

The environmental distributions of *Lactobacillus* and *Bifidobacterium* species are highly diverse. Although some species exclusively populate in specific niches (e.g. *Lactobacillus delbrueckii* in dairy products and *Lactobacillus johnsonii* and *Bifidobacterium bifidum* in the gastrointestinal tract), others are found in various environments (e.g. *Lactobacillus plantarum* and *Lactobacillus casei*) [26, 27]. It should be mentioned that because of the extreme diversity at phenotypic, ecological and genotypic levels among species within the genus *Lactobacillus*, the genus has been recently reclassified into 25 genera, including *Lactobacillus* (the *L. delbrueckii* group, as previously referred to), *Paralactobacillus* and 23 novel genera [28]. Here, we still use the previous classification that regards the genus *Lactobacillus* as a whole, given that the probiotic *Lactobacillus* species that are analysed in the following sections are defined according to it. The differences in the niche adaptability of these probiotic species suggest that at least three kinds of adopted lifestyles exist: free-living (food habitat), nomadic and strictly symbiotic. Because the associations between the bacteria (*Lactobacillus* and *Bifidobacterium*) and the hosts or food-associated habitats are ancient [29–31], the bacteria may exhibit different genomic features that reflect their fitness in various niches due to long-term co-evolution.

For example, milk-adapted *Lactobacillus bulgaricus* has undergone genome decay [32] and expresses specific enzymes to forage typical milk-derived sugars [33]. Moreover, the *B. bifidum* species contains the genetic elements involved in processing mucin-derived carbohydrates, which are believed to be important during gut colonization [34, 35]. The nomadic *L. plantarum* contains a ‘lifestyle adaptation’ genomic cassette that enables the use of a diverse set of sugars [36]; the species also has a relatively high number of regulatory functions, and shows the absence of certain genomic signatures [26] to adapt itself to diverse habitats. Nevertheless, such conclusions are derived on the basis of single-genome sequence analysis or genome comparisons between several strains from different niches, making them informative only for a narrow taxonomic window and not applicable for rational strain selection.

To identify the genome features that determine the different gut colonization potential of probiotic species, we initially collected thousands of genome assemblies and BioSample information on 19 species of *Lactobacillus* and *Bifidobacterium* to reveal their different adopted lifestyles via comparative genomics analysis. The chosen species were all permitted food additives according to the Chinese National Health Commission, the US Food and Drug Administration (FDA) and the European Food Safety Authority (EFSA), and are commonly found in commercial products worldwide. We analysed and correlated the guanine–cytosine (GC) content, genome size, phylogenetic relationship, intraspecies genetic diversity and branching order of the 19 species. In addition, we compared the profiles of carbohydrate-using enzymes (especially those responsible for mucin cleavage), the presence or absence of bile salt hydrolase (BSH) genes and the corresponding sequence dissimilarity, and the distribution of genes that are considered to be typical colonization factors (e.g. pilus and S-layer protein) between the species. Furthermore, for the nomadic species, the niche-specific genes were identified. Finally, we tried to distinguish the strains by lifestyle using all these colonization-related genetic elements as inputs.

## Methods

### Genome sequences and niche information

A total of 1665 sequenced genomes (Table S1) belonging to 14 species of *Lactobacillus* (*L. sakei*, *L. delbrueckii*, *L. salivarius*, *L. gasseri*, *L. crispatus*, *L. johnsonii*, *L. casei*, *L. acidophilus*, *L. plantarum*, *L. reuteri*, *L. rhamnosus*, *L. paracasei*, *L. fermentum* and *L. helveticus*) and 5 species of *Bifidobacterium* (*B. breve*, *B. bifidum*, *B. longum*, *B. adolescentis* and *B. animalis*) were retrieved from the Refseq and GenBank databases of the National Center for Biotechnology Information (NCBI). All available genomes of the species in the database were downloaded unbiasedly on 11 December 2018, and five abnormal assemblies were removed due to their significant genetic distance from the other strains in their separate species-level phylogenetic tree (Fig. S1) and misclassification (Table S2). The niche information for each strain was obtained from the BioSample database (NCBI), and the data concerning genome size, whole-genome GC content and number of CDSs were also retrieved.

### Single-nucleotide polymorphism (SNP) identification, phylogeny reconstruction, assembly annotation, pan-genome analysis and recombination identification

The SNPs of all included sequenced genomes were identified as previously described [37]. In brief, each genome was mapped to the reference genome of the corresponding species using MUMmer (Table S1, each individual reference genome of each species is in bold) [38], and then the obtained bi-allelic SNPs in the core genome were combined into an SNP matrix for each species. A neighbour-joining tree was built for each species based on the sequences of the concatenated SNPs using the Treebest tool (http://treesoft.sourceforge.net/treebest.shtml). The assemblies were reannotated using Prokka [39], and the annotated results were served as inputs to conduct pan-genome and gene presence/absence analyses (with a minimum blastp percentage identity of 90 %) using Roary [40]. The core genes of each species were defined by those present in 99–100 % of the strains. A phylogenetic tree reflecting occurrence time was built using reference 16S rRNA gene sequences of 14 *Lactobacillus* and 4 outgroup species (*Bacillus coagulans*, *Bacillus subtilis*, *Bacillus vallismortis* and *Enterococcus faecalis*). The outgroup is considered to be ‘one or more of the species that are assumed to fall outside of the species group of interest (denoted the ingroup)’ and it is generally accepted that ‘the branch where the outgroup connects to the ingroup becomes the root of the ingroup tree’ [41]. Here, four outgroup species were selected according to a previous report [42]. For inferring the root of this phylogenetic tree, the outgroup method – which is the most commonly used one and is expected to be a powerful method for rooting phylogenetic trees [41, 43, 44] – was used; the tree was rooted using the branch leading to the outgroup species according to previous reports [41, 45]. The software ClonalFrameML based on maximum likelihood (ML) method was used to identify bacterial recombination [46]. The genome alignment for specific species and the corresponding ML tree constructed by raxml-ng [47] were used as input files. The non-core regions were ignored during calculation. The relative effect of recombination over mutation was equal to *r*/*m*=(*R*/*θ*) × *δ* × *ν* [48]. *R*/*θ* is the ratio of recombination and mutation rates, *δ* is the average tract length of a recombination event and *ν* is the rate of new polymorphisms introduced by recombination.

### Taxonomic characterization of reclassified BSHs

The construction of protein databases of BSH and annotation of BSH genes were conducted as previously reported [49]. In brief, the protein databases of BSH were constructed by collecting sequences from the Refseq database using the keywords ‘bile salt hydrolase’ and ‘choloylglycine hydrolase’. The protein sequences of individual genomes from Prokka were taken as a query against the above reference protein database by using blastp with an E-value of 1e-5, sequence identity of 45 % and reference coverage of 50 % as cutoff. Then 24 BSH reference sequences from the HMP database were added to achieve a final collection of 2282 BSH sequences (Table S3). The range of sequence length was confined between 300 and 400 aa bp. The sequence alignment was conducted by MAFFT [50], and a neighbour-joining tree based on the BSH sequences was built using Treebest (http://treesoft.sourceforge.net/treebest.shtml). The alignment of typical BSH sequences was visualized using BioEdit.

### Enzyme profiles involved in carbohydrate metabolism

We chose four types of enzymes, namely glycoside hydrolases (GHs), glycosyltransferases (GTs), polysaccharide lyases (PLs) and surface layer homology domains (SLHs), to represent the overall profiles of carbohydrate-utilizing modules according to previous reports [45, 51–53]. These enzymes were predicted across the 1665 genomes by using the HMMSCAN [from the HMMER package 3.1b2 (http://hmmer.org/)] to query the hidden Markov model-based CAZyme dbCAN database according to a previously described approach [45]. We chose a cutoff of 50 % coverage and E-value <1e-5 for protein sequences beyond 80 aa, and 50 % coverage and E-value <1e-3 for protein sequences below 80 aa. For host-derived carbohydrate use, given that gut colonization by bacteria is reported to be correlated with their ability of using host-derived glycans (mucin glycans) [35, 54, 55], we chose the nine enzymes [chitinase (EC 3.2.1.14), neuraminidase/sialidase (EC 3.2.1.18), α-galactosidase (EC 3.2.1.22), beta-galactosidase (EC 3.2.1.23), α-N-acetylgalactosaminidase (EC 3.2.1.49), α-N-acetylglucosaminidase (EC 3.2.1.50), α-l-fucosidase (EC 3.2.1.51), beta-N-hexosaminidase (EC 3.2.1.52) and Endo-α-N-acetylgalactosaminidase (EC 3.2.1.97)] that have been nominated to be involved in mucin glycan utilization in a recent study [56] to evaluate the distribution of these enzymes among the analysed probiotic species/strains. The reference proteins were retrieved from the Refseq database by using the names of mucin glycan-processing enzymes as keywords. The protein sequences of individual genome from Prokka were taken as queries against the above reference protein database by using blastp with an E-value of 1e-5, reference coverage of 50 % and sequence identity of 45 % as a cutoff.

### Niche-specific variation detection within each nomadic *Lactobacillus* species

For each nomadic *Lactobacillus* species, gene-based and SNP-based genome-wide association studies (GWASs) without correction for population structure were used to identify niche-specific genes/variants within the species according to a previously reported approach [57]. pyseer software was used [58]. Since pyseer only supported continuous or binary phenotypes, the binary phenotype pair, human-origin vs free-living, was selected. The source information ‘commercial dietary supplements’, ‘commercial probiotic’, ‘Infloran capsule’ and ‘probiotic sachet’ were excluded because they did not represent the natural niche of the strains. The phenotypes of animal sources were also discarded. The gene presence/absence table from Roary and SNP presence/absence table converted from preliminary SNP matrix were used. Genes or SNPs found in 1–99 % of the total population were retained. The fixed model was chosen for both SNP and gene analysis. The significance threshold for each analysis was set using Bonferroni correction with a required *P*-value of 0.05/number of tested variants. The visualization of GWAS results was achieved by drawing Manhattan plots using the qqman package in R. For the species that the pyseer fed back null model, the analysis was not included.

### Typical colonization factors

Some bacterial genes, known as gut colonization factors, such as mucus-binding protein [13], S-layer protein [59], pili [17, 60], luxS [18, 19], serine-rich glycoprotein adhesion [61] and antimicrobial peptide [62], which have been reported to be important for host–microbe interactions, were selected to evaluate their distribution among the analysed species/strains. Separate protein databases were constructed by collecting sequences from the Refseq database and/or the Uniprot database using the keywords ‘mucus-binding protein’, ‘S-layer protein’, pili (‘LPXTG’, ‘sortase’, and ‘pilus, fimbria and fimbrial protein’), ‘luxS’ and ‘serine-rich glycoprotein adhesin.’ The Antimicrobial Peptide Database (APD) was used directly (http://aps.unmc.edu/AP/) according to a previously described approach [63]. The protein sequences of individual genomes from Prokka were taken as a query against the above reference protein database by using blastp with an E-value of 1e-5, sequence identity of 45% and reference coverage of 50 % as a cutoff.

### Cluster of Orthologous Groups of proteins (COG) annotation and distinction of the three lifestyles

According to a previously reported method [52], COG function categories for genes were annotated by blastp amino acid sequences against a COG database (version 2014) with a threshold of 45 % identity, 50 % query coverage and an E-value of 1e-10. The genes below the threshold were discarded from our analysis. All genomic features of 1665 genomes, including genome size, GC content and numbers of BSHs, eight mucin cleavage enzymes, LPXTG, sortase, pilus, luxS, mucus-binding protein, S-layer protein, serine-rich glycoprotein adhesin and antimicrobial peptide, were used as inputs to conduct principal component analysis (PCA) using the prcomp function in R software. The PCA plot was visualized using the ggbiplot package in R. PCA was also conducted using COG functions as inputs.

## Results

### Three lifestyles among different species and their phylogenetic structures

**Fig. 1.**
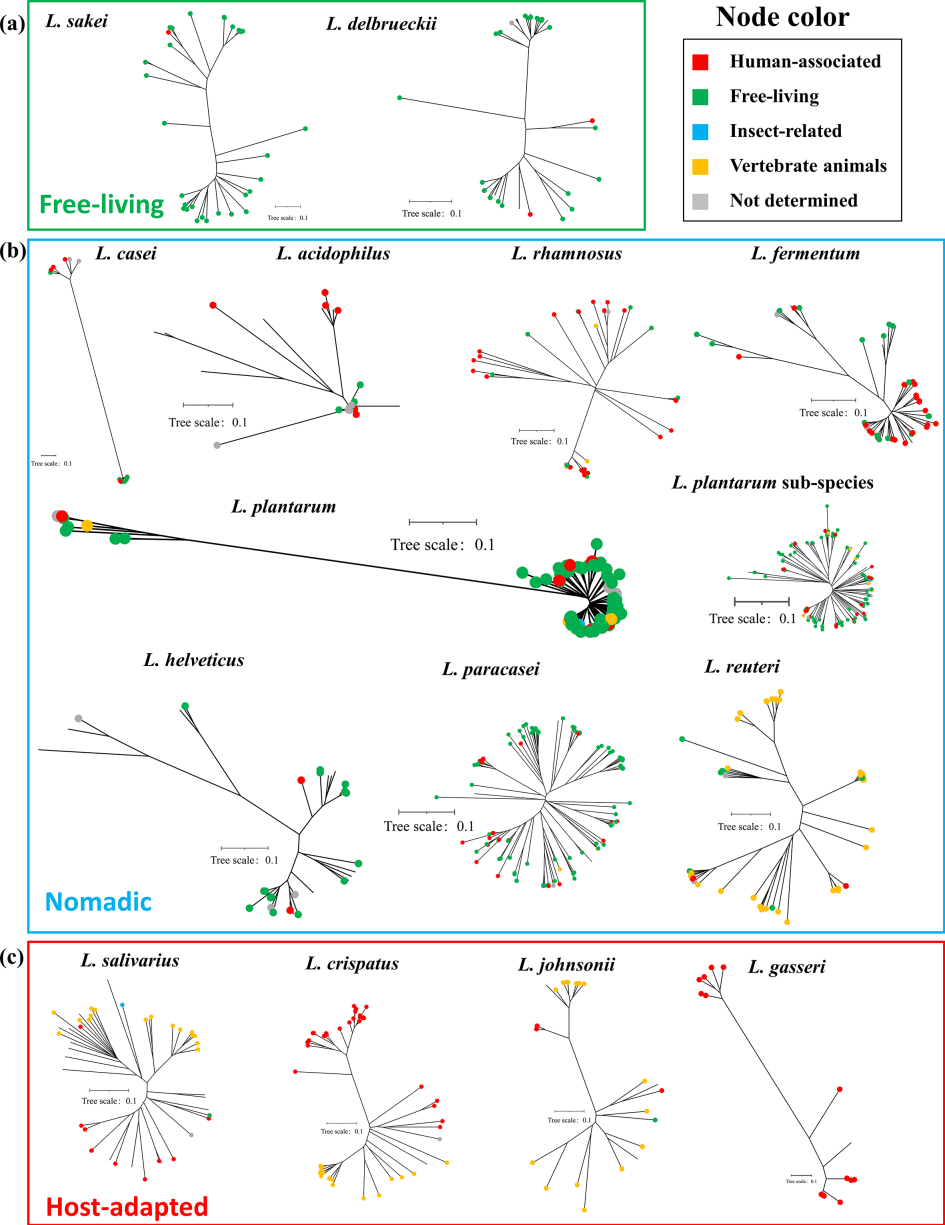
*Lactobacillus* species covered three lifestyles, namely free-living (**a**), nomadic (**b**) and strictly symbiotic (**c**). The phylogenetic trees of the 14 *Lactobacillus* species are shown, and the population structure of the dominant *L. plantarum* sub-species is also presented. Niche information is represented by the coloured nodes of each tree. Niches are categorized into human-associated habitats (red), free-living environments such as food niches or plants (green), insect-related niches (blue), vertebrate animals (orange) and pure bacterial cultures or commercial supplements in which the isolation origins of microbial strains cannot be determined (grey). The branch that does not have nodes represents those strains without niche information in the NCBI database.

**Fig. 2.**
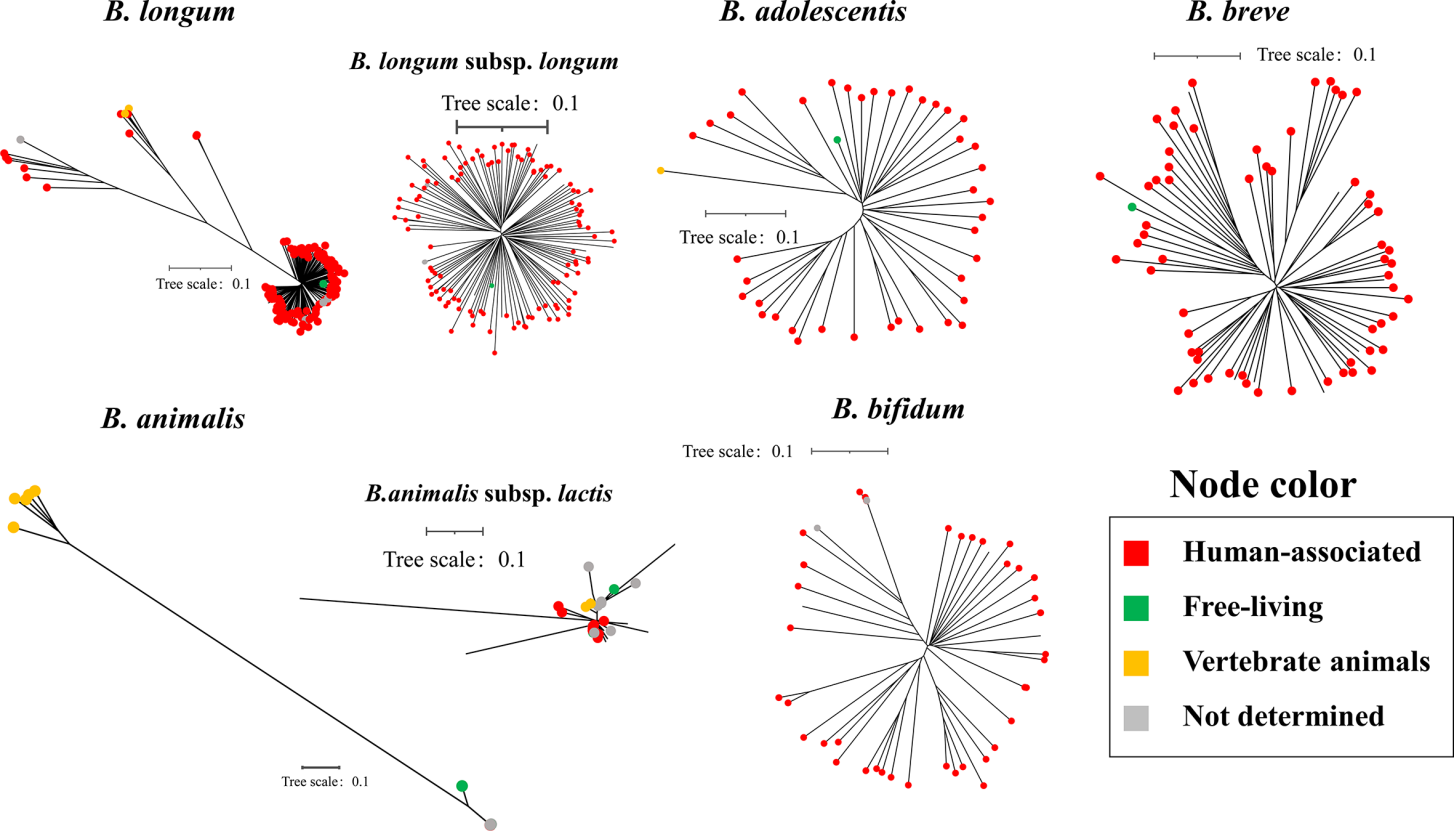
*Bifidobacterium* species showed niche conservatism, tended to be host-adapted, and exhibited a high recombination population structure. The phylogenetic trees of the five *Bifidobacterium* species are shown, and the population structures of the dominant *B. longum* and *B. animalis* subspecies are presented. Niche information is represented by the coloured nodes of each tree. Niches are categorized into human-associated habitats (red), free-living environments such as food niches or plants (green), vertebrate animals (orange), and pure bacteria cultures or commercial supplements in which the isolation origins of microbial strains cannot be determined (grey). The branch that does not have nodes represents those strains without niche information in the NCBI database.

The lifestyle modes of the 19 species of bacteria were accessed by summarizing all available isolation origins of the 1665 corresponding strains that have genome assemblies in the NCBI database (Figs 1, 2, S2 and S3). We collected the niche data for 1343 of the 1665 strains (80.7 %) (Table S1). We found that the *Lactobacillus* strains covered three lifestyle modes (Figs 1a−c and S2a, c). Such lifestyle diversity of *Lactobacillus* can be partly explained by its greater genomic diversity [45]. Recently, the genus *Lactobacillus* has been reclassified into 25 genera due to marked phenotypic, ecological and genotypic differences among species [28]. Our results indicated that most *Lactobacillus* species analysed in this study were in the nomadic mode. Notably, *L. plantarum* could be encountered in extremely diverse niches. Although they were nomadic, *L. reuteri* were relatively constrained to two kinds of niches and exhibited a preference for animals (78.4 %, especially in the forestomach of mice) over humans (7.8 %), and for sourdough (6.0 %) over other food niches (2.6 %) in the current database. For host-adapted *Lactobacillus* species, we found that *L. salivarius*, *L. crispatus* and *L. johnsonii* were promiscuously host-adapted, and that *L. gasseri* were specifically human-adapted. Moreover, we found that *L. johnsonii* were more likely to be isolated from animals, in particular mice, which corresponded with the 16S rRNA gene sequencing data that indicated its dominance in the mouse forestomach [64]. Lastly, we found that the free-living *L. delbrueckii* (88.6 %) and *L. sakei* (96.8 %) strains were more frequently present in food/plant niches or other environmental sites, and were only occasionally host-associated.

All five species of *Bifidobacterium* were host-adapted bacteria, with few strains found in food niches and the environment (Figs 2 and S3). Notably, accurate discrimination of subspecies of *B. longum* via genome-based phylogenetic tree enabled us to match niches across four sub-phylotypes, and its colonization mode showed a subspecies-specific lifestyle.

The phylogenetic structures of the 19 species constructed based on SNPs in their core-genomes revealed varied patterns among the species (Figs 1 and 2). For *Bifidobacterium*, the phylogenetic trees of *B. breve* [recombination/mutation (r/m): 1.44], *B. adolescentis* (r/m: 2.41), *B. bifidum* (r/m: 4.14) and *B. longum* subsp. *longum* (r/m: 3.72) exhibited ‘radiation’ or ‘fuzzy’ shapes, indicating that these population structures had undergone recombination that disturbed the vertical genetic signal [65]. For *Lactobacillus*, the phylogenetic patterns were relatively diverse.

### General genomic features, intraspecies genetic diversity and branching order

**Fig. 3.**
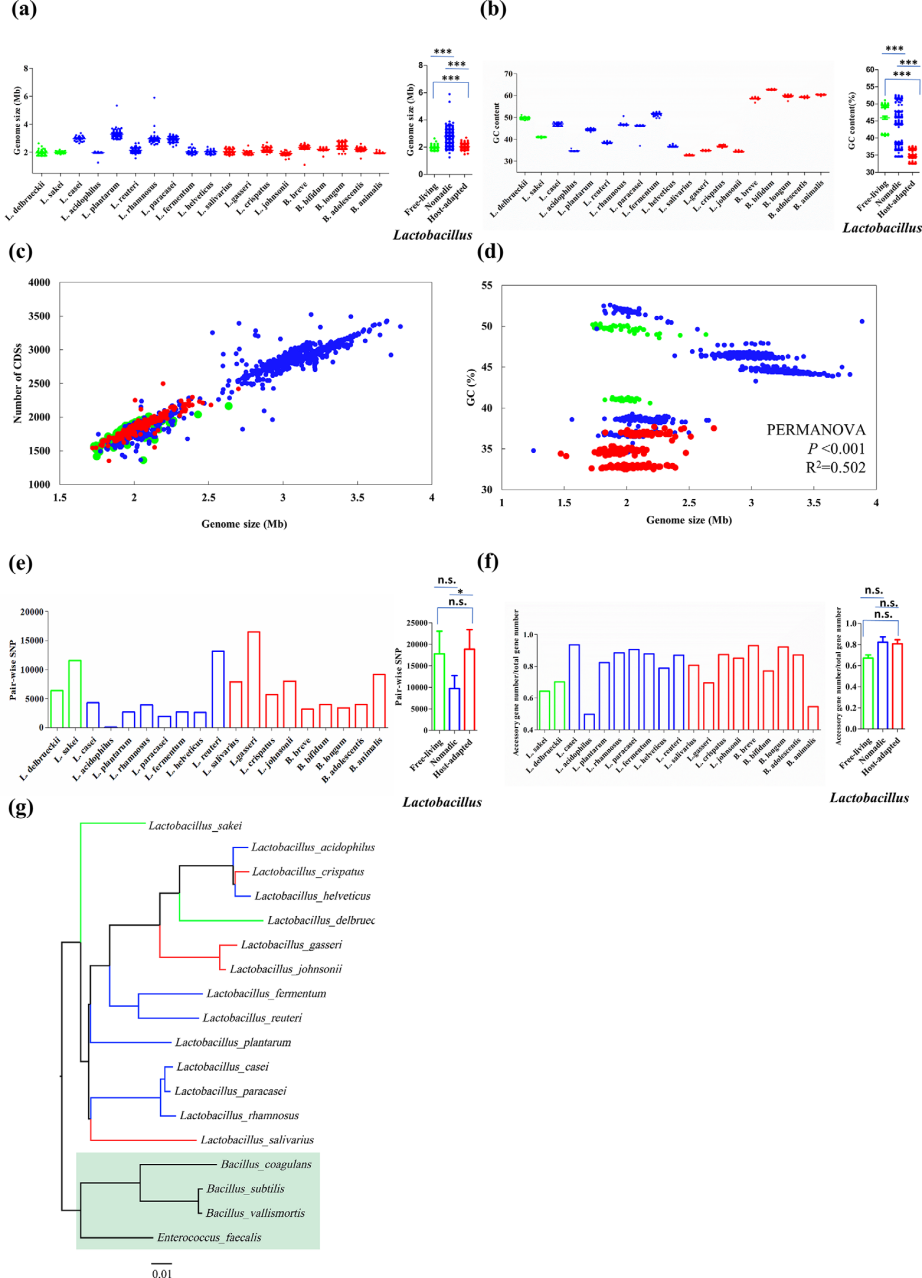
Lifestyle variations of *Lactobacillus* species correlated with the separation of general genomic features, demonstrated by reduction of GC content and genome size from free-living to nomadic, and finally to host-adapted lifestyle. (**a**) Comparison of genome size (Mb) by species (left) and by lifestyle (right, only for *Lactobacillus*). (**b**) Comparison of GC content (%) by species (left) and by lifestyle (right, only for *Lactobacillus*). (**c**) Association between genome size (Mb) and the number of CDSs for *Lactobacillus* species (1212 genomes included), Pearson *R*=0.98, *P*<0.0001. (**d**) Association between genome size (Mb) and GC content (%) for *Lactobacillus* species (1182 genome included), Pearson *R*=0.51, *P*<0.0001. (**e, f**) Comparison of intraspecies genome diversity by SNP distance (**e**) and accessory genome size (**f**) by species (left) and by lifestyle (right, only for *Lactobacillus*). Average pairwise SNP distance among strains of each species was calculated and normalized (divided) by their respective genome size. (**g**) Phylogenetic tree constructed using reference 16 s rRNA gene sequences of the 14 *Lactobacillus* species and rooted using the branch leading to 4 outgroup species (*Bacillus coagulans*, *Bacillus subtilis*, *Bacillus vallismortis* and *Enterococcus faecalis*). The outgroup species are highlighted in the green background. The bars for significance are from the comparisons between *Lactobacillus* lifestyles. Mann–Whitney U test: ***, *P*<0.001; *, *P*<0.05; ns, *P* >0.05.

The genome size and GC content of the 1665 strains were analysed (Fig. 3a, b). We found that the nomadic *Lactobacillus* species tended to possess larger genome sizes, highlighted by the typical nomadic species *L. casei*, *L. plantarum*, *L. rhamnosus* and *L. paracasei*, while host-adapted lactobacilli had relatively reduced genomes (host-adapted lactobacilli versus nomadic lactobacilli: *P*<0.001, Mann–Whitney U test). These results were consistent with those reported by a review in terms of genome analysis focusing on a reference strain of individual lactobacillus species [16], in which the trend of genome reduction along the lifestyle transition route was proposed. We also observed a reduction in the GC content in host-adapted *Lactobacillus* species compared with nomadic species (*P*<0.001, Mann–Whitney U test) and free-living species (*P*<0.001, Mann–Whitney U test), in line with one well-documented A (adenine)- and T (thymine)-enriched mutational pattern following the non-adaptive loss of DNA repair genes during the genomic evolution of symbionts [66]. The genome size correlated linearly with the number of coding sequences (CDSs) for *Lactobacilli* species of the three living modes (Fig. 3c). Notably, by using the two-dimensional features of GC content and genome size, the preliminary separation of *Lactobacillus* species of three lifestyles were shown (PERMANOVA: *P*<0.001; Fig. 3d). We found that the *Bifidobacterium* species exhibited similar GC content and genome size, which might be an outcome of adaptations to a host lifestyle.

Further insight was obtained by analysing the intraspecies genetic diversity in the background of SNP variations in the core genomes and the ratio of accessory genes (Figs 3e, f and S4a, b). We found that the host-adapted *Lactobacillus* species showed a larger intraspecies genetic distance (more numbers of average pair-wise SNP) compared with nomadic ones (*P*<0.05, Mann-Whitney U test). One notable exception was *L. reuteri*. It was a nomadic species but exhibited remarkably high intra-species SNP variations. This observation might be explained by its dominant residence in host environments (86.2%). Notably, it has been reported that *L. reuteri* has diversified into host-specific lineages and represented distinct ecotypes reflecting adaptation to different vertebrates [25, 67–69]. We further analysed the distribution of SNP distance in strain-level instead of the above addressed species-level average SNP distance, confirming that nomadic species represented lower genomic diversity (Fig. S4a). The genetic diversity in terms of the accessory gene number/total gene number ratios did not differ between *Lactobacillus* strains of the three lifestyles (*P*>0.05, Mann-Whitney U test). *L. acidophilus* and *B. animalis* represented lower accessory genome sizes. The accessory gene number/total gene number ratios seemed not to be obviously affected by included genomes for each species when different proportions of genomes were sampled (Fig. S4b). The *L. acidophilus* had the lowest intra-species diversity with respect to both intraspecies SNP variations and accessory gene numbers, making it unusual among *Lactobacillus* species, which needs further research.

As for branching order (occurrence time), a phylogenetic tree was constructed based on reference 16S rRNA gene sequences of each species with the evolutionary ancestors of the genus *Lactobacillus* as outgroup (*Bacillus coagulans*, *Bacillus subtilis*, *Bacillus vallismortis*, and *Enterococcus faecalis*) [42] (Fig. 3g). It has been reported that the branch that is positioned closer to the outgroup suggests members of this branch might exist earlier than the other branches [51, 70]. Our results indicated that *L. sakei* belonged to a separate clade close to outgroup, which suggests that it might exist earlier than the other species. The other 13 *Lactobacillus* species were located in parallel clades, which cannot reflect the branching order.

### Reclassification and variation patterns of BSHs

Bacterial bile salt hydrolase (BSH; EC 3.5.1.24), also known as cholylglycine hydrolase, leads to the hydrolysis of conjugated bile salts into deconjugated bile acids, and thus protects the bacteria in the gut. BSH has been found in several bacterial genera, such as *Lactobacillus* [71, 72], *Bifidobacterium* [73], *Enterococcus* [74], *Clostridium* spp. [75], and *Bacteroides* [76]. BSH has paralogues within individual strains [77, 78], showing high levels of sequence dissimilarity and consequently, functional heterogeneity.

**Fig. 4.**
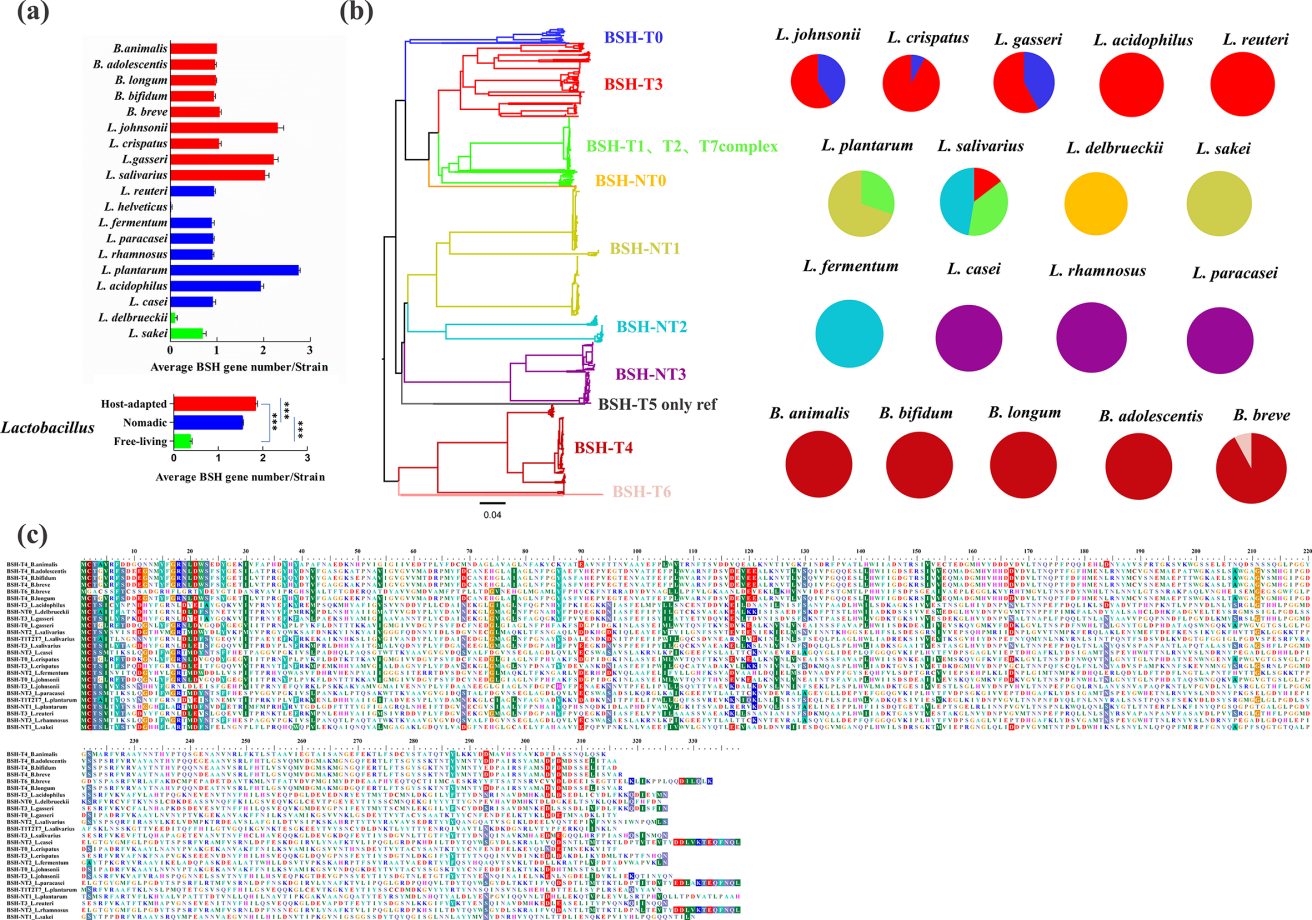
Copy numbers and phylotypes of BSH genes showed marked dissimilarity between the three bacterial lifestyles, and four new phylotypes were identified that had not previously been encountered in the gut microbiome. (**a**) Average BSH gene number per strain for each species (up) and each lifestyle (down, only for *Lactobacillus*). (**b**) Reclassified BSH subtypes from reconstructing phylogeny with 2282 BSH sequences from *Lactobacillus* and *Bifidobacterium* strains in addition to the reference BSH sequences from the human gut microbiome (see Table S2 for details), and the distribution of these BSH types by lifestyle. (**c**) Alignment of typical BSH sequences. The bars for significance are from the comparisons between *Lactobacillus* lifestyles. Mann–Whitney U test: ***, *P*<0.001.

We looked up the BSH genes to strain level to determine whether the activity and numbers of BSH genes were distinguishable by lifestyle (Fig. 4a, Table S3). The results revealed that only one strain of *L. helveticus* showed a BSH gene (1/54). We found that the host-adapted lactobacilli tended to have more BSH genes (usually >one paralogue in one strain) than the nomadic and free-living lactobacilli, with the exceptions of *L. acidophilus* and *L. plantarum*. The host-adapted *Lactobacillus* species harboured a significantly higher number of BSHs than the nomadic and the free-living *Lactobacillus* species (Mann–Whitney U test: *P*<0.001 and *P*<0.001), and the nomadic lactobacilli also possessed a markedly higher number of BSHs than the free-living ones (Mann–Whitney U test: *P*<0.001).

Further information comes from the phylogenetic analysis of the identified BSHs. As shown in Fig. 4b, the BSHs of the *Bifidobacterium* species were exclusively categorized into the BSH-T4 type, in line with the results from the gut microbiome [49]. However, *B. breve* showed a small part of BSH-T6 activity. As reported, BSH-T3 showed the most stable complex and enzymatic activity among BSH-T0 to BSH-T7 [49], and in line with this, all host-adapted *Lactobacillus* species in this work showed T3 activity. The free-living *L. delbrueckii* and *L. sakei*, and the nomadic *L. fermentum*, *L. casei*, *L. rhamnosus* and *L. paracasei*, exclusively possessed new types of BSHs that had not previously been encountered in the gut microbiome. The sequence dissimilarity was further visualized by the alignment of representative BSH paralogues identified in each species (Fig. 4c).

### Distribution of carbohydrate utilizing enzymes and in particular mucin-foraging ability

**Fig. 5.**
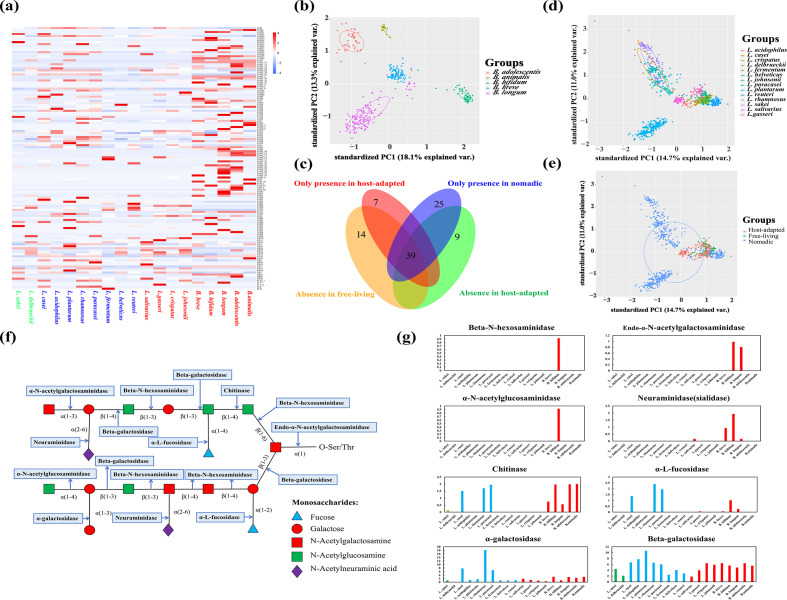
Nomadic *Lactobacillus* species had more diverse carbohydrate-utilizing profiles, and *Bifidobacterium* demonstrated broad host-derived glycan-foraging ability, in particular O-linked glycan-degrading activity. (**a**) Heatmap of the average number of GTs, GHs, PLs and SLHs per strain among the various species. (**b**) PCA plot of the sugar-utilizing enzyme profiles of the *Bifidobacterium* species. (**c**) Venn plot of niche-associated sugar-utilizing genes. (**d, e**) PCA plot of the sugar-utilizing enzyme profiles of the *Lactobacillus* at the species (**d**) or lifestyle levels (**e**). (**f**) Illustration of nine mucin glycan-cleaving enzymes. (**g**) Mucin glycan foraging ability among the three bacterial lifestyles (gene number/strain). Species from left to right: *L. sakei*, *L. delbrueckii*, *L. casei*, *L. acidophilus*, *L. plantarum*, *L. rhamnosus*, *L. paracasei*, *L. fermentum*, *L. helveticus*, *L. reuteri*, *L. salivarius*, *L. gasseri*, *L. crispatus*, *L. johnsonii*, *B. breve*, *B. bifidum*, *B. longum*, *B. adolescentis* and *B. animalis*.

One of the main factors that determines bacterial adaptability in their habitats is nutrient availability. We next investigated the profiles of the carbohydrate-utilizing enzymes of our bacterial species, emphasizing their host-derived mucin glycan-foraging abilities. The average numbers of GHs, (GTs, PLs and SLHs in each species are shown in Fig. 5a. We found that the *Bifidobacterium* species encoded several GHs that *Lactobacilli* did not (members of GH13, GH30, GH43, GH5 and GH50) or rarely encoded (members of GH43, GH51 and GH94). When looking in detail at the carbohydrate metabolism capabilities of *Bifidobacterium* species at the strain level, we found that *B. longum* showed a relatively large intraspecies difference, while the others showed metabolic conservatism (Fig. 5b).

We then explored the difference in carbohydrate-utilizing enzymes in the *Lactobacillus* species with the three lifestyles (Fig. 5c). Nomadic lactobacilli harboured more diverse GHs and GTs, in line with their adaptability in a broad range of habitats. We defined that if one given gene family is present in at least one species of certain lifestyle, the status of the gene family for the lifestyle is ‘presence’; otherwise, when the gene family is absent from all the strains of certain lifestyle, the status is called ‘absence’. In total, 39 gene families were shared among the *Lactobacillus* species with the 3 lifestyles, 25 were exclusively present in the nomadic strains and 7 were exclusively found in the host-associated species. Fourteen gene families were absent in the free-living lactobacilli, and nine showed loss in the host-adapted bacteria. This set of niche-associated GTs or GHs included a few interesting genes worth highlighting. GH33 (encompassing exo-sialidases), involved in host-glycan degradation, only appeared in the host-adapted *Lactobacillus* species. Other GHs encoding metabolic ability on host-derived glycans, such as GH20 (including activities of hexosaminidase), GH125 and GH38 (representing α-mannosidases activities) and GH29 (involving in fucosidases), were absent in the free-living lactobacilli. In addition, GH51 and GH137, which were reported to separately encode plant-derived dietary fibre-degrading α-l-arabinofuranosidases and β-l-arabinofuranosidases [79], were only found in the host-adapted lactobacilli. Further, the strain-level distribution of these metabolic genes indicated that the host-adapted and free-living lactobacilli that resided in relatively constant niches (gut or fermented food) harboured more stable carbohydrate metabolism profiles, while the nomadic *Lactobacillus* species reflected highly diverse metabolic signatures (Fig. 5d, e).

Benefiting from nine enzymes nominated in a recent study that can split mucin glycans into oligo- and monosaccharides as well as separating glycans from mucin proteins (Fig. 5f) [56], we evaluated the distribution of these mucin glycan-degrading enzymes among *Bifidobacterium* and *Lactobacillus* species/strains. As shown in Fig. 5g, α-N-acetylgalactosaminidase was not encountered in the genomes of all included *Lactobacillus* and *Bifidobacterium* strains. *B. bifidum* was a fascinating example of mucin degraders with all remaining eight enzymes, and such activity seemed to be a species-level property. The ability to utilize O-linked glycans (catalysed by endo-α-N-acetylgalactosaminidase − with which bacteria could cleave glycans from mucin proteins) was confined to *B. bifidum* and *B. longum*. All five species of *Bifidobacterium* showed chitinase activity, and paralogues of this enzyme were found in *B. bifidum*, *B. adolescentis* and *B. animalis*. The *Lactobacillus* species also harboured the potential to degrade mucin glycans because they all exhibited α-galactosidase and beta-galactosidase activities, and *L. salivarius* stood out due to its sialidase activity. The free-living lactobacilli did not have α-l-fucosidase genes.

### Niche-specific variations

**Fig. 6.**
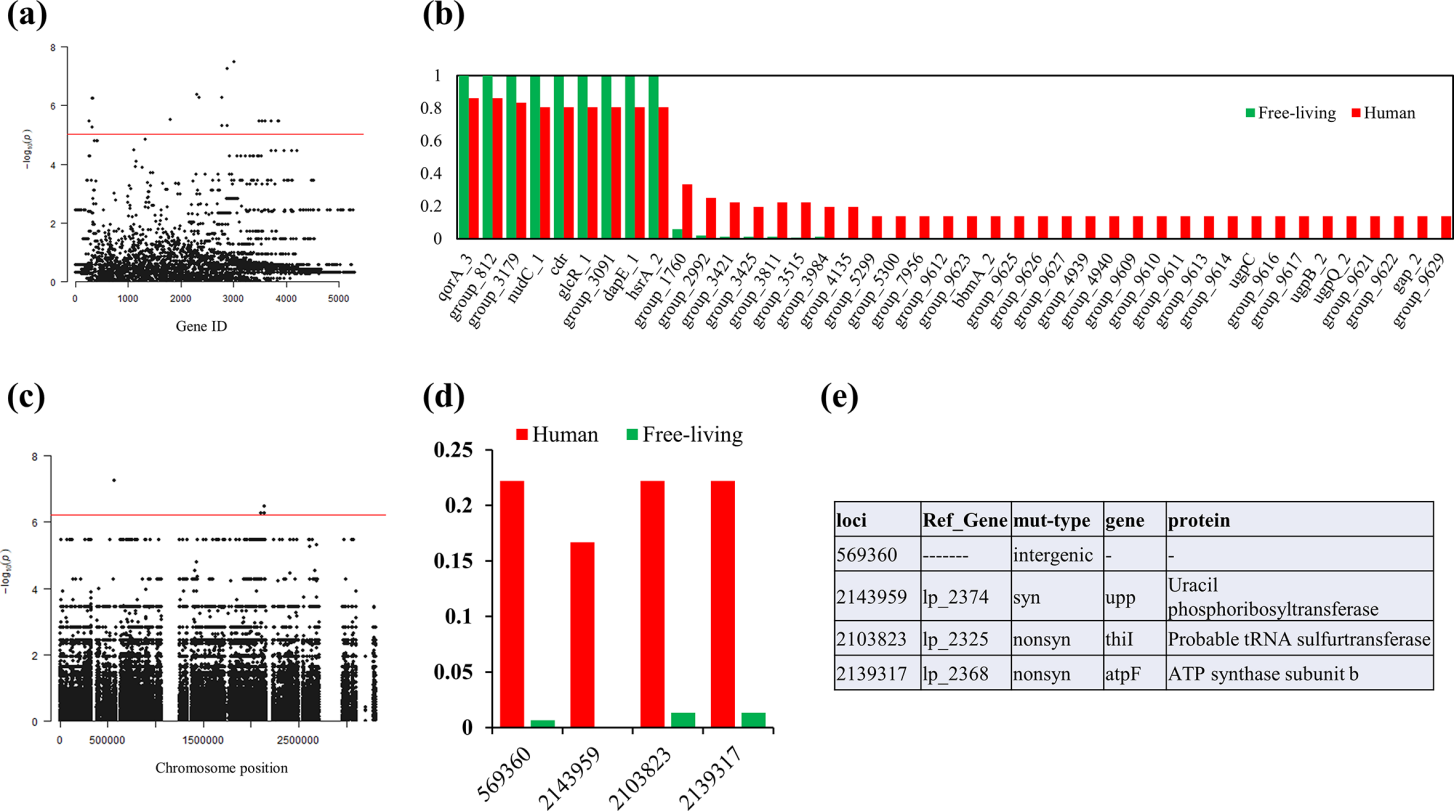
Human-adapted genes and variations stood out in nomadic *L. plantarum* revealed by genome-wide association study (GWAS) analysis. (**a**) Manhattan map shows genes with significant correlations with the human niche. Significance threshold is marked by the horizontal line. Genes were arranged along the *x*-axis according to the random order. (**b**) Occurrence frequency of the marked genes in bacterial strains of the two separate niches (the number of strains with this gene/total number of strains in the given niche). (**c**) Manhattan map shows the SNPs with significant correlations with the human niche. Significance threshold is marked by he horizontal line. (**d**) Occurrence frequency of the marked SNPs in bacterial strains of the two separate niches (the number of strains with this SNP status/total number of strains in the given niche). (**e**) Annotation of significant SNP loci.

Next, we tried to identify the sets of genes and SNP variations that were differentially present in distinct niches for each of the nomadic species, respectively. Forty-two genes stood out in the association for *L. plantarum* (Fig. 6a, b). These genes can be categorized into three types: genes that were unique to the human niche, genes that were found predominantly in humans and genes that were harboured by all free-living strains but represented absence in strains of human origin (Fig. 6b). This set of significant hits included a few interesting genes worth highlighting. Nine genes involved in carbohydrate-related metabolism and transport (maltose and l-arabinose related: bbmA_2, group_9626, group_9627, group_4939, gap_2 and group_9617; glycerol-3-phosphate import: ugpC, group_9616, ugpB_2, and ugpQ_2) were only detected in the human-associated strains. Another marked feature was the enrichment of gene integration elements (group_9614 and group_1760) in the strains of human origin. It was believed that the presence of integration elements might contribute to more frequent horizontal gene transfers, and such gene elements were considered to be drivers for genome evolution [80, 81]. In contrast, human-adapted *L. plantarum* strains seemed to exhibit an ongoing loss of genes for regulation (glcR_1). In the relatively stable gut niche, some regulatory functions of bacteria might be redundant in comparison to more elastic environments [82]. No significant hits were obtained for *L. rhamnosus*, *L. paracasei* and *L. fermentum* (Fig. S5a). However, one hit was very close to the significance threshold for *L. rhamnosus*, one hypothetical protein (group_1903), and this gene was more likely to be present in the free-living niche.

We also tested the SNP variation differences in the core genomes between the strains of the two niches, and detected four SNPs for *L. plantarum* (Fig. 6c−e). These included two that caused nonsynonymous mutation (located in genes thil and atpF, respectively), one in the intergenic region and one that encoded synonymous mutation (located in gene upp). The fluctuated pathways or biological processes included UMP biosynthesis via the salvage pathway, cofactor biosynthesis and substrate transportation. We did not obtain significant hits for *L. rhamnosus*, *L. paracasei* and *L. fermentum* (Fig. S5b). However, one SNP located in the LGG_RS10130 gene (encoding alpha-glucosidase) stood out from all the others, although it was below the significance line. This SNP locus was apparently differentially distributed among the *L. rhamnosus* strains of the two niches.

### Colonization factors

**Fig. 7.**
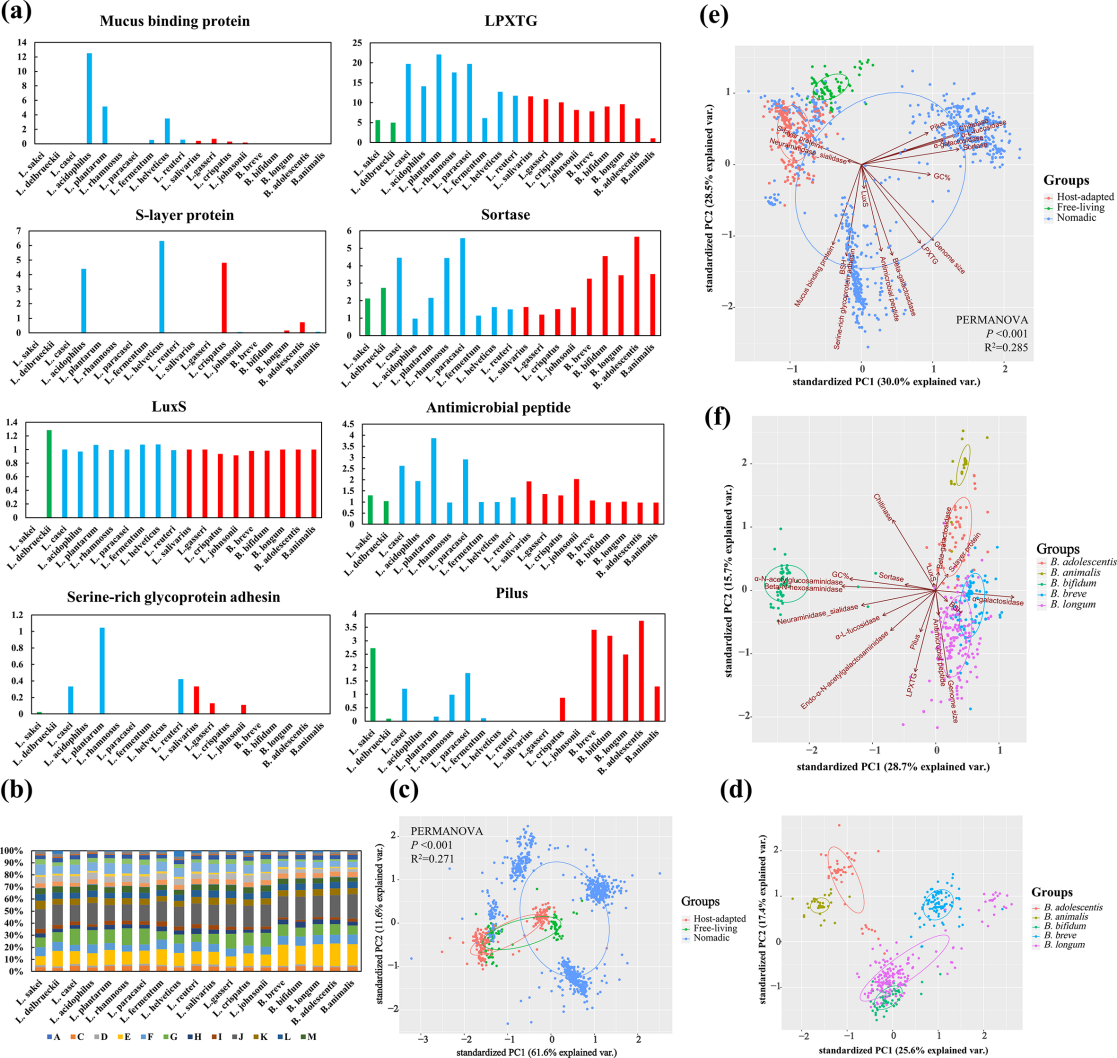
Species with three lifestyles were better separated using gut colonization factors compared with COG functions. (**a**) Distribution of reported gut colonization genes by lifestyle (gene number per strain for each species). (**b**) Distribution of COG functions by lifestyle. (**c**) PCA plot of COG functions of *Lactobacillus* among the three lifestyles. (**d**) PCA plot of COG functions of the various *Bifidobacterium* species. (**e**) PCA plot of the gut colonization-related genes of *Lactobacillus* among the three lifestyles. (**f**) PCA plot of gut colonization-related genes of the different *Bifidobacterium* species.

Several *Lactobacillus* and *Bifidobacterium* genes or molecules have been proposed to mediate host–microbe interactions based on the established functions of these genes in host adhesion and the results of intra-species comparative genomic analysis using small sample sets [13, 17–19, 59–62], despite their limitations, and we can still further explore the distribution modes of these colonization factors among different species. It was evident that these colonization-associated genes (apart from the BSH and mucin-foraging enzymes described earlier) could be divided into two categories: intestinal tissue-anchored elements (e.g. mucus-binding protein) and signalling molecules (e.g. luxS gene and antimicrobial peptides). The distribution of these colonization factors among the various bacterial species of the three lifestyles are illustrated in Fig. 7a. Bacterial surface proteins, including mucus-binding protein, S-layer proteins and serine-rich glycoprotein adhesion, were largely absent in the free-living *Lactobacillus* species, but were enriched in some species of the nomadic and host-adapted *Lactobacillus*. Pilus structure, together with LPXTG and sortase element, showed a broad coverage among the 19 species. Signal substance-related genes also showed a broad coverage among the species, with the exception of *L. sakei*, which had no luxS genes. The luxS gene has been shown in *B. breve* UCC2003 [19] and *L. reuteri* 100–23 [18] to be involved in the production of the interspecies signalling molecule autoinducer-2 (AI-2), which promotes biofilm formation in the digestive tract, and thus mediates bacterial colonization.

Long-term co-evolution of bacteria with their natural niche may yield functional separation between species in various environments. We asked whether species of the three lifestyles could be distinguished by their annotated functions. The relative percentages of average COG categories in each species and the PCA results based on the COG categories are presented in Fig. 7b−d. Preliminary separation was demonstrated between *Lactobacillus* species of the three lifestyles (PERMANOVA: *P*<0.001, *R* ^2^=0.271; Fig. 7c). For all host-adapted *Bifidobacterium* species, *B. longum* and *B. bifidum* exhibited similar functional imprints, while *B. longum* showed the greatest intra-species diversity (Fig. 7d). We noticed that our separation method using the genetic features of colonization-associated molecules generally achieved comparable segregation (PERMANOVA: *P*<0.001, *R* ^2^=0.285; Fig. 7e). The different clustering behaviours of the nomadic species indicated that a more detailed category system is needed. Using these colonization-related genetic features, *B. longum*, *B. breve* and *B. adolescentis* clustered more closely, whereas *B. animalis* and *B. bifidum* were independent (Fig. 7f).

## Discussion

The driving concept of this study was that the genomic features of bacteria reflect their lifestyles and indicate their gut colonization potential, whether such traits are the outcomes of long-term ecological selection or neutral evolution. The features mined here are largely selected from the published literature in which these genes or enzymes have been proved to be important for mediating host–microbe interactions [13, 17–20, 35, 54, 55, 59–62]. Notably, causal associations between some of these features and gut colonization by probiotic bacteria have been validated via molecular methods. For example, inactivation of luxS in *L. reuteri* 100–23C caused an increase in the thickness of the biofilm formed *in vivo* and affected its ecological performance [18]; heterologous expression of bile salt hydrolase genes (bshA from *L. acidophilus* NCFM and bshB from *L. johnsonii* NCK88) in *Escherichia coli* C600 significantly increased colonization biomass in the faeces of germ-free mice compared with that of control *E. coli* C600 [20]. On the other hand, species from distinct niches had different gut fitness, in which strains of lactobacilli that are autochthonous to the human gut showed better gut colonization ability after oral ingestion compared with the allochthonous lactobacilli [83]. Therefore, we firmly believe that the features described here are not just consequences of the lifestyle of these taxa rather than their potential ability to colonize the gut.

Our screen of 1665 strains of 19 species of *Lactobacillus* and *Bifidobacterium* yielded a number of interesting features. For example, we found that the host-adapted lactobacilli tended to have lower GC content, as was pointed out by one well-documented pattern of GC reduction during genome evolution of symbionts [66]. Other potentially interesting genetic features that have not been reported previously include e.g. the near absence of BSH genes in the *Lactobacillus* species, *L. helveticus* (54 genomes included), which is widely used in traditional fermented food; the recombination-characterized population structure of the *Bifidobacterium* species and genus-level host adaptability; the enrichment of some carbohydrate enzymes targeting to plant-derived polysaccharide (e.g. l-arabinose) in the host-adapted clade of the nomadic *L. plantarum* species; the remarkable interspecies sequence dissimilarity of BSH genes; and the presence of four new types of BSHs that have not previously been encountered in the gut microbiome.

Benefitting from the vast statistical analysis on bacterial niche information, we further defined the three lifestyles of *Lactobacillus* and *Bifidobacterium* species and categorized the 19 probiotic species with clinical application potential accordingly. Although the classification of *Lactobacillus* lifestyle has been reported by a review focusing on reference strains of individual *Lactobacillus* species [16], our results did reinforce the significance of definition. In this paper, we reclassified *L. reuteri* into nomadic lifestyle. It should be mentioned that *L. reuteri* was previously considered to be a host-adapted species, which ignored its common occurrence in sourdough. Frequent isolation of *L. reuteri* from food niches as previously reported [84, 85] supported the view that persistence of the species in fermented food is not occasional. Metagenomic data of human gut microbiota supported the free-living modes of *L. delbrueckii* and *L. sakei*, since *L. delbrueckii* was not detected in the human faecal samples, and *L. sakei* could only be detected in very low abundance at the first time point but could not persist in longitudinal samples [86].

Due to possible sampling biases from isolating these bacteria, the species belonging to free-living and host-adapted lifestyles were likely to be reclassified into nomadic modes, if considerable isolates of these species from the other type of niche (either food niches/environment or host gut) were observed or their occurrence was detected via metagenomic analysis in future. For example, for host-adapted *Lactobacillus* species, few food-derived isolates have been reported in the NCBI database, and it is possible that a reasonable quantity of new strains will be isolated from or stable occurrence of the species will be detected in food or environmental niches besides the currently identified host gut, and the species will be thus reclassified as nomadic. For *Bifidobacterium*, the conclusion tended to be independent of sampling biases, because the strictly anaerobic nature of *Bifidobacterium* species determined their association with hosts to avoid oxygen pressure. Notably, *B. longum* represented a subspecies-specific lifestyle, which reinforced previous reports that *B. longum* subsp. *longum* is dominant in the human gut throughout the human lifespan [24], *B. longum* subsp. *infantis* is more likely to be isolated from human infants [87], and *B. longum* subsp. *suis* and the newly identified *B. longum* subsp. *suillum* are frequently found in piglets [88].

In addition, we observed species-level population structures of *Bifidobacterium* species affected by recombination among strains. Recombination and point mutations are two major impetuses of bacterial evolution [89]. Recombination leads to the replacement of DNA short chunks with the homologous segments from other strains, and contributes more to diversification compared with point mutations [25]. Recombination is considered to be an important host-driven evolution factor for *L. reuteri* and plays a crucial role in its host adaptability [25].

Our conclusions have the potential to guide probiotic selection, improving host health status in a chosen manner. The *Bifidobacterium* species and host-adapted *Lactobacillus* species should be selected as a priority if gut colonization is the main aim to lastingly modulate the indigenous microbiome, or to function as a metabolic factory; in other conditions, where immunomodulatory effects should be emphasized, free-living strains are proposed. Further, it is possible that certain nomadic lactobacilli might show better ecological fitness compared with some host-adapted ones, inspired by the fact that a majority of *L. plantarum* (296/310) demonstrated marked advantages in intestinal tissue-anchored surface structures (serine-rich glycoprotein adhesin).

Another message conveyed by our data was that the distribution of gene elements that accounts for colonization phenotypes was not only species-specific, but also strain-specific. Around 16 % of *L. salivarius* had sialidase, 1 % of *L. fermentum* were without BSH, more than 66 % of *L. reuteri* lost mucus-binding proteins, and various strains of *L. plantarum* harboured one to three BSH paralogues. Such strain-specificity in the genome content provided an explanation for the previously reported intraspecies colonization discrepancies [13, 90]. Further insight came from niche-specific genes or variations within the most exemplified nomadic *Lactobacillus* species, *L. plantarum*, which was identified using an unsupervised method. The identified human-specific bacterial genes and SNPs would be directly used to infer the true niche of bacteria and even to predict the colonization phenotype.

Our results highlighted that nutrient availability might be closely correlated with the lifestyles of bacteria, and that it is one of the main factors determining bacterial adaptability in their habitats. We found that the *Bifidobacterium* species encoded several GHs that *Lactobacilli* did not (members of GH13, GH30, GH43, GH5 and GH50) or rarely encoded (members of GH43, GH51 and GH94). In line with our results, the genus *Bifidobacterium* was reported to harbour one of the largest collections of GH13, GH43 and GH51 family members (2.0, 2.6 and 7.0 fold more than the average GH repository of the gut microbiome, respectively), together with the *Bacteroides* spp. and family *Clostridiales* [91]. Families GH13, GH43, GH51 and GH5 harboured similar degradative abilities for complex plant-derived polysaccharides [92]. This suggests that the *Bifidobacterium* species may outcompete other microbiota for undigested plant-derived dietary fibre in the gut. Furthermore, members of GH13 (which account for 32.9 % of extracellular GHs), GH43 (24 %) and GH51 families (12 %) are typical putative extracellular enzymes [91], which can also benefit the host in accessing dietary fibres.

For *Lactobacillus*, the GHs encoding metabolic ability on host-derived glycans, such as GH20, GH125, GH38 and GH29, were absent in the free-living lactobacilli, which might support the notion that food niche-adapted microbial species tend to undergo genome decay to discard redundant genes, especially sugar-utilizing enzymes, when adapting to specific habitats [32]. In addition, GH51 and GH137, which were reported to encode plant-derived dietary fibre-degrading enzymes [79], were only found in the host-adapted lactobacilli, an observation suggesting co-evolution and metabolic interactions between host, diet and gut symbionts. Furthermore, nine genes involved in carbohydrate-related metabolism and transport were only detected in the human-associated strains of *L. plantarum* (representative nomadic species). This can be explained by different sugar profiles in fermented food and the human gut, where maltose and l-arabinose corresponded separately with starch-based staple food and cellulose- and pectin-enriched dietary fibres in the gut, possibly suggesting bacterial adaptation to the host’s diet.

Further insight comes from mucin glycan-utilizing enzymes. It was observed that *Bifidobacterium* species have broad enzyme profiles pointing to mucin glycans, in particular O-linked glycans, which might be an important contributor to its host-adapted lifestyle; for *Lactobacillus* species, the host-adapted lactobacilli did not show any advantages in either gene types or counts of such enzymes over the strains of the other two lifestyles, indicating that mucin-foraging ability might not be a necessary factor for *Lactobacillus* to determine colonization phenotypes.

The strains of the three lifestyles could be separated with a collection of colonization-related genomic content. This suggests that we could embrace such complexity of bacterial colonization using complete bacterial genomic information. A very recent review proposed determination of the natural niche of bacteria using their positions in the phylogenetic tree [16], yet this approach is likely to be ineffective because the strains with the same niche did not cluster (Figs 7 and S5). Moreover, the phylogenetic tree based on the core genome can only reflect kinship but ignores the information on the accessory gene sets. Indeed, exactly these accessory genes largely determine the ecological fitness. Therefore, in future, a method should be proposed to use the genome contents as variables, possibly the colonization factors mined in this study, to predict bacterial engraftment or their niche in nature if sufficient colonization data in humans or model animals are available. Following the concept we proposed in our review article [93], prediction can be based on a machine-learning algorithm, such as gradient-boosting regression, random forest and neural networks. Taking the gradient-boosting regression algorithm [94] as an example, a two-stage approach (a discovery stage and a validation stage) can be employed. The discovery and validation datasets should be used independently, and a leave-one-out cross-validation tactic can be adopted for model training. The model can be based on gradient-boosting regression and predict the niche of strains using the sum of thousands of different decision trees. The algorithm infers all the trees sequentially and trains each tree on the residual of all previous trees with an accumulated contribution to the overall prediction. Each tree contains the specific features that represent the genomic properties of microbes. To reveal factors underlying prediction, the relative importance of each feature can be analysed via partial dependence examination. Different algorithms/approaches for such niche or engraftment prediction will be compared, and under further evaluation via various data sets.

## Supplementary Data

Supplementary material 1

Supplementary material 2
