# Supplementary material for: Mining genome traits that determine the different gut colonization potential of Lactobacillus and Bifidobacterium species
Source: Microb Genom. 2021 Jun 8;7(6):000581. doi: 10.1099/mgen.0.000581 (PMC8461469; doi:10.1099/mgen.0.000581)
Supplement: Supplementary material 1 [file mgen-7-0581-s001.pdf]

Supplemental Materials for:

Mining genome traits that determine different gut colonization potentials of *Lactobacillus* and *Bifidobacterium* species

Yue Xiao[a,b], Jianxin Zhao[a,b], Hao Zhang[a,b,c,d,f] , Qixiao Zhai[a,b,e*], Wei Chen [a, b, c, g]

[a] State Key Laboratory of Food Science and Technology, Jiangnan University, Wuxi, Jiangsu 214122, People's Republic of China

[b] School of Food Science and Technology, Jiangnan University, Wuxi, Jiangsu 214122, China

[c] National Engineering Research Center for Functional Food, Jiangnan University, Wuxi, Jiangsu 214122, China

[d] (Yangzhou) Institute of Food Biotechnology, Jiangnan University, Yangzhou 225004, China

[e] International Joint Research Laboratory for Probiotics at Jiangnan University, Wuxi, Jiangsu 214122, China

[f]Wuxi Translational Medicine Research Center and Jiangsu Translational Medicine Research Institute Wuxi Branch

[g]Beijing Innovation Centre of Food Nutrition and Human Health, Beijing Technology and Business University (BTBU), Beijing 100048, P.R. China

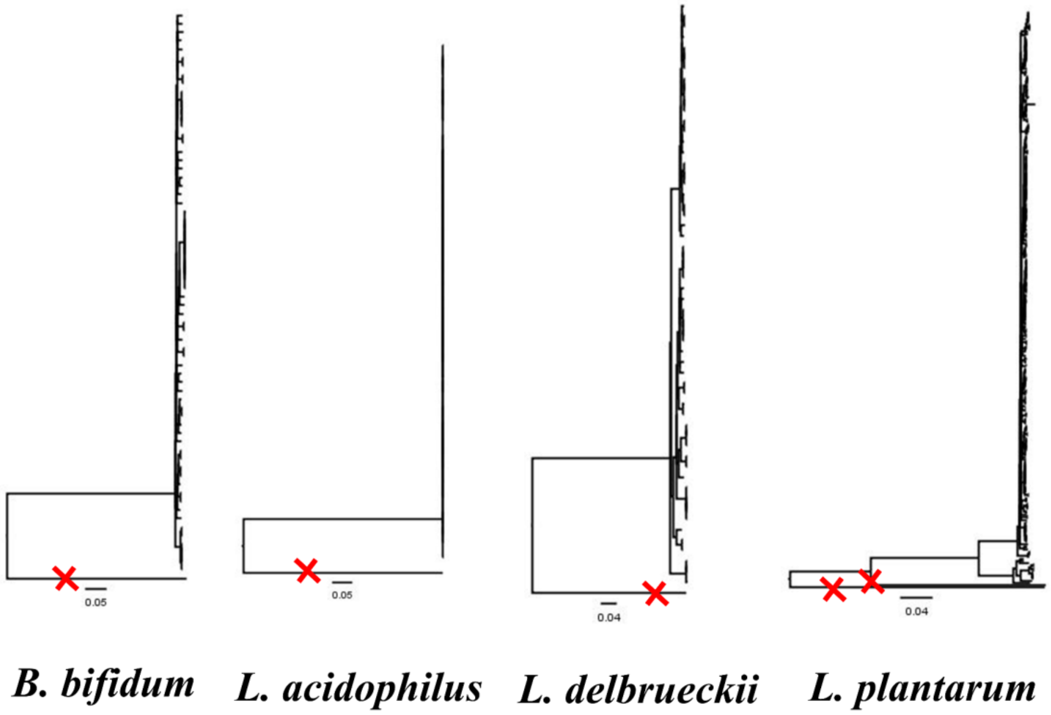

Figure S1 The phylogenetic trees for *Lactobacillus* and *Bifidobacterium* species with abnormal assemblies. The obvious outliers were marked with red cross.

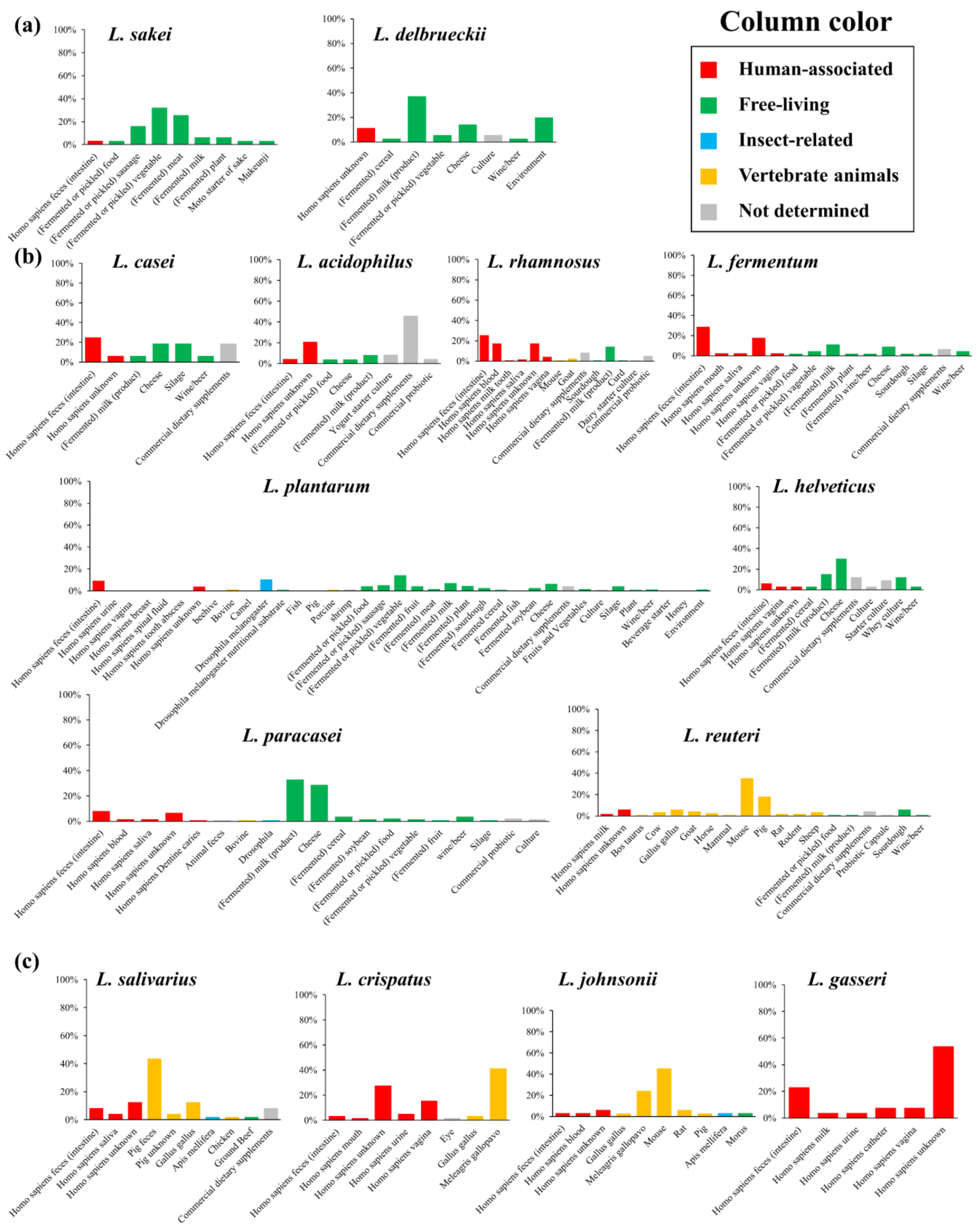

Figure S2 The summarized niche information obtained from the NCBI BioSample database for strains of 14 *Lactobacillus* species. The raw niche information was represented by each column

without technical curation in order to capture more accurate and detailed outline of niche distribution. Niches were categorized into the human-associated habitats (red), free-living environments such as food matrices or plants (green), insect-related niches (blue), vertebrate animals (orange), and pure bacterial cultures or commercial supplements in which the isolation origins of microbial strains cannot be determined (gray).

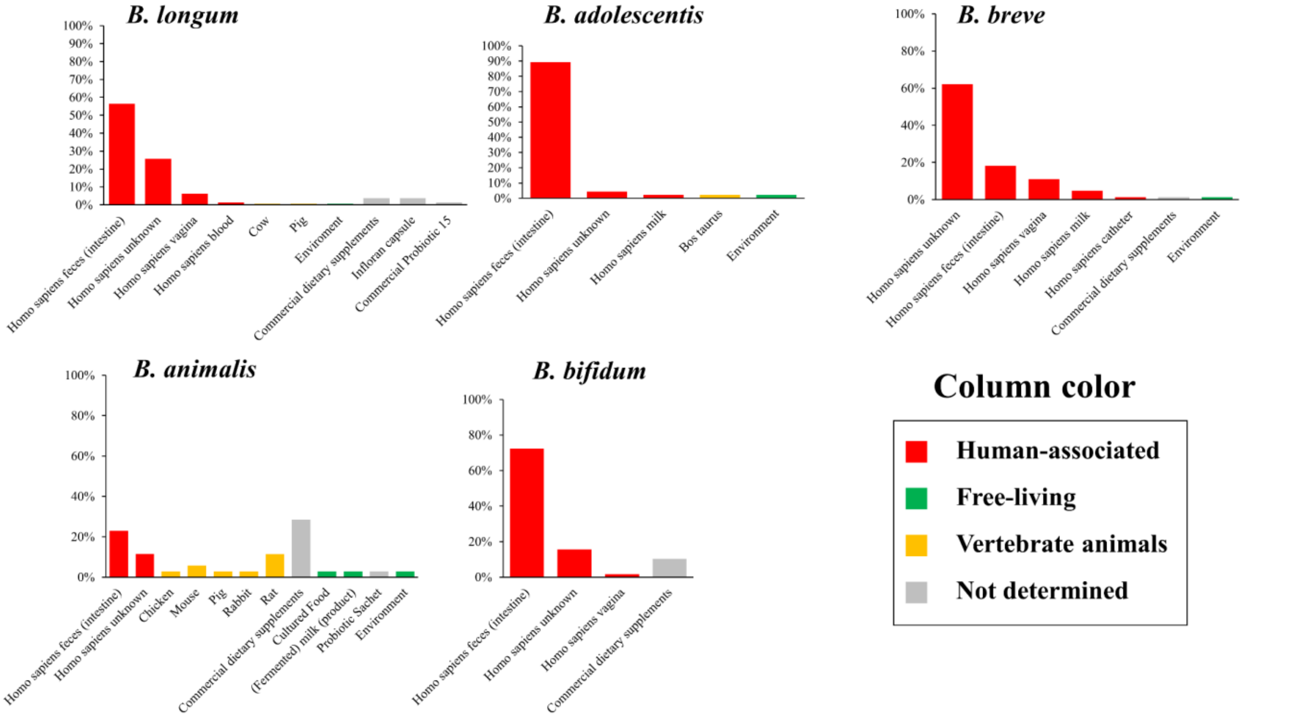

Figure S3 The summarized niche information obtained from the NCBI BioSample database for strains of 5 *Bifidobacterium* species. The raw niche information was represented by each column without technical curation in order to capture more accurate and detailed outline of niche distribution. Niches were categorized into the human-associated habitats (red), free-living environments such as food matrices or plants (green), insect-related niches (blue), vertebrate animals (orange), and pure bacterial cultures or commercial supplements in which the isolation origins of microbial strains cannot be determined (gray).

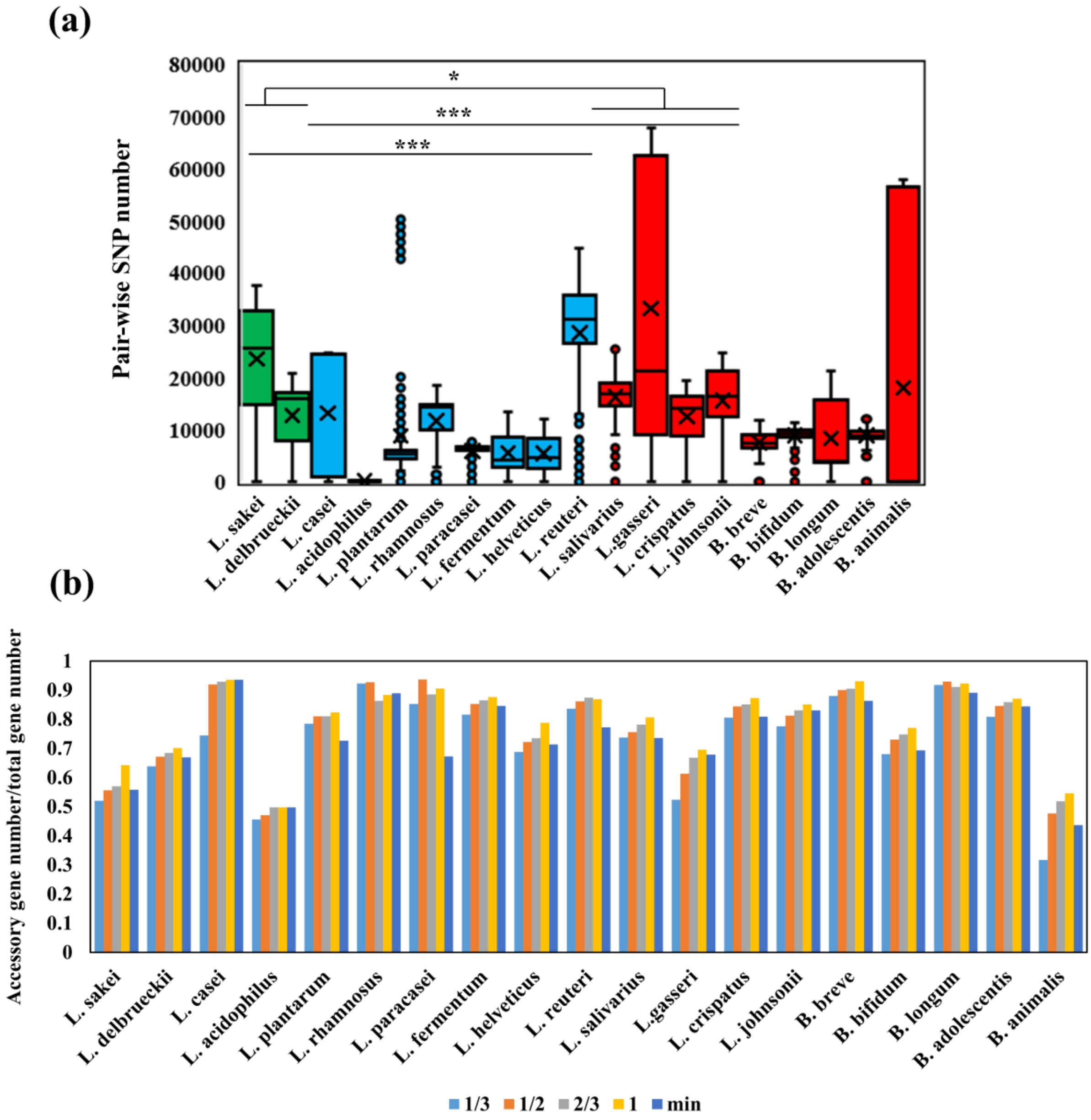

Figure S4 Intra-species genomic diversity of different *Lactobacillus* and *Bifidobacterium* species. (a) Pair-wise SNP distance between any two strains within each species was plotted. (b) The ratio of accessory gene number/total gene number for each species. Different proportions (numbers) of genomes were sampled, in which 1/3, 1/2, and all of the total available sequenced genomes for each species were used. “min” was 24 that represented the minimum analyzed genomes (the number of genomes for *L. casei*) among 19 species. Mann-Whitney U test: “*”, *P* <0.05; “***”, *P* <0.001.

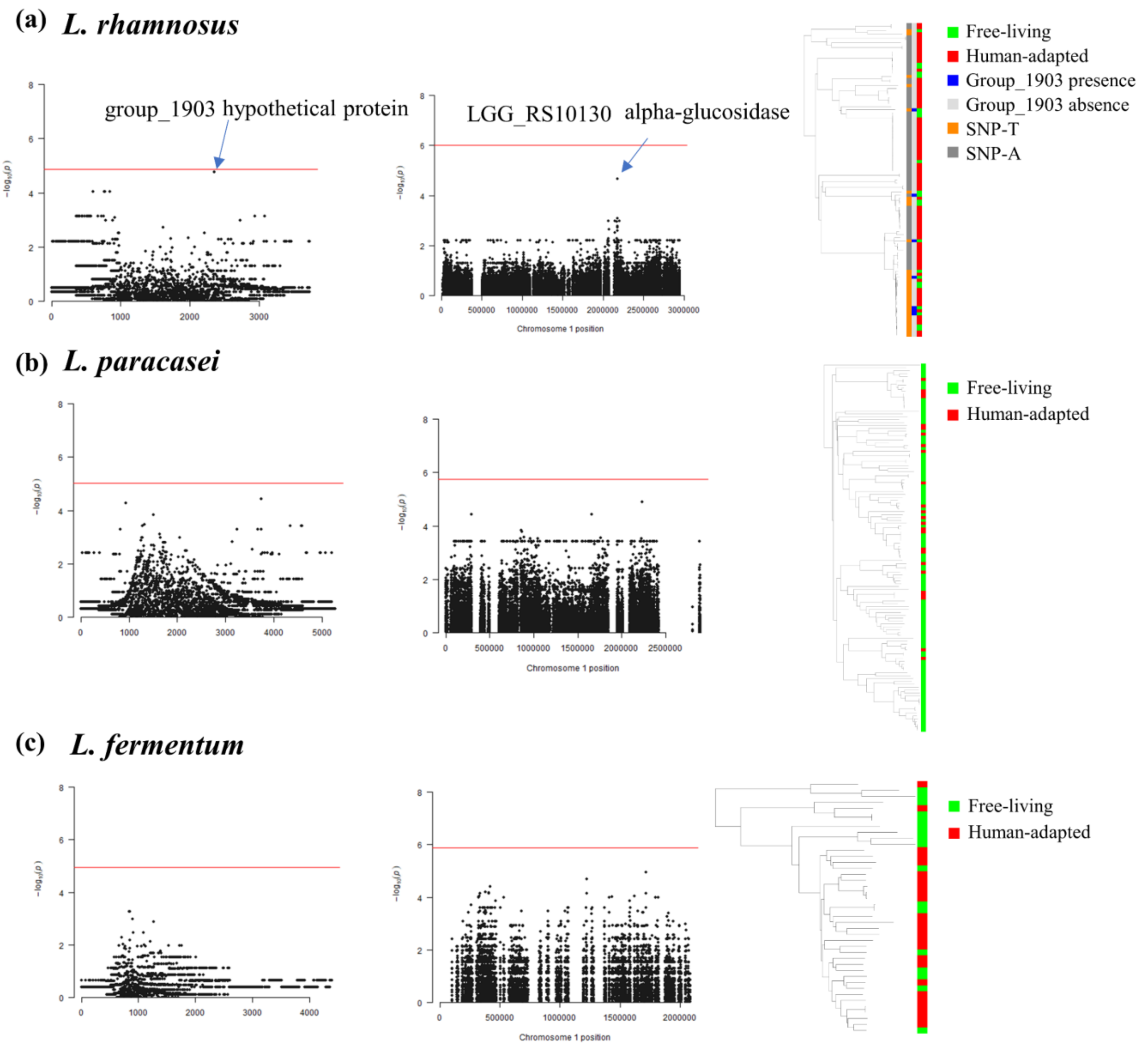

Figure S5 Nomadic species *L. rhamnosus*, *L. paracasei*, and *L. fermentum* demonstrated no significant signals for niche-specific genes or SNPs. (a) Manhattan map of genes (left) and SNPs (middle) after GWAS analysis. Significance threshold is marked by the horizontal line. Distribution of outlier genes and SNPs across the phylogenetic tree and the two niches (right) for *L. rhamnosus*. (b and c) Manhattan map of genes (left) and SNPs (middle) after GWAS analysis with the significance threshold marked by the horizontal line. Distribution of niche information across the phylogenetic tree (right) for *L. paracasei* (b) and *L. fermentum*(c).
